# Initial experience with single-port robotic hysterectomy

**DOI:** 10.1590/S1679-45082017AO4134

**Published:** 2017

**Authors:** Mariano Tamura Vieira Gomes, Andréa Maria Novaes Machado, Sérgio Podgaec, Gustavo Anderman Silva Barison

**Affiliations:** 1 Hospital Israelita Albert Einstein, São Paulo, SP, Brazil.; 2 Hospital das Clínicas, Faculdade de Medicina, Universidade de São Paulo, São Paulo, SP, Brazil.

**Keywords:** Hysterectomy, Myoma, Minimally invasive surgical procedures/methods, Gynecologic surgical procedures/methods, Uterine neoplasms, Adenomyosis, Single-port, Histerectomia, Mioma, Procedimentos cirúrgicos minimamente invasivos/métodos, Procedimentos cirúrgicos em ginecologia/métodos, Neoplasias uterinas/cirurgia, Adenomiose, Portal único

## Abstract

**Objective:**

This article presents the first series of robotic single-port hysterectomy cases performed at a hospital in Brazil.

**Methods:**

From November 2014 to October 2016, 11 patients were indicated to undergo, and nine of them were submitted to single-port hysterectomy using da Vinci Single-Site^®^ platform. However, in two patients, due to multiple previous abdominal surgeries, large uterine volume, and/or a uterus with no mobility, a pneumoperitoneum was performed with a Verres needle, and the pelvic cavity was assessed using a 5mm optics endoscope. In these cases, single-port surgery was not recommended; therefore, multiportal robotic access was chosen, and no intercurrent events were reported. Nine single-port cases were operated on by the same surgeon at *Hospital Israelita Albert Einstein.* Patient data analyzed included age, body mass index, previous surgeries, and clinical diagnosis. Surgical data included operative time, skin incision, report of intraoperative complications, need for conversion to laparotomy, need for transfer to intensive care unit, need for blood transfusion, inadvertent injury to other organs, length of hospital stay, and death.

**Results:**

All cases were completed with da Vinci Single-Site^®^ system, with no intercurrent events. Four patients presented with adenomyosis as the surgical indication, two had uterine myoma, one endometrial cancer, one endometrial polyp, and one desquamative inflammatory vaginitis. The mean age of patients was 44 years (range, 40 to 54 years), and body mass index varied between 23.4 and 33.2kg/m^2^ (mean 26.4). No complications occurred in any of the cases, such as intestinal or bladder injury, bleeding, or the need for a second surgery. All nine procedures were completed with the robotic single-port access, and no patient required a blood transfusion.

**Conclusion:**

Although this study merely presented an initial series of patients submitted to robotic single-port surgery, it demonstrated that the method is feasible and safe, suggesting the possible use of this technique in elective hysterectomy and other gynecological procedures in the future, as described in large reference centers of advanced surgery worldwide. Specifically, in gynecological practice, existing evidence on the use of robot-assisted, single-port surgery seems promising, and although it is not indicated in all cases, it should be considered as a surgical option. Nonetheless, further randomized and controlled clinical studies are necessary to establish the preeminence of robot-assisted, single-port surgery *versus* single-incision and conventional laparoscopy.

## RESUMO

**Objetivo:**

Apresentar a primeira série de casos de histerectomia usando sistema robótico de portal único *(single-port)* em hospital no Brasil.

**Métodos:**

No período de novembro de 2014 a outubro 2016, de modo inédito no Brasil, 11 pacientes tiveram indicação inicial e 9 delas foram submetidas à histerectomia por portal único, com a plataforma da Vinci Single-Site^®^. Em duas pacientes, devido a múltiplas cirurgias abdominais prévias, grande volume uterino e/ou útero sem mobilidade, optou-se pela instalação de pneumoperitônio com agulha de Verres e inspeção da cavidade pélvica com ótica de 5mm, constatando-se, nestes casos, não ser viável a cirurgia por *single-port*, levando-se, assim, à opção pela técnica robótica multiportal, sem intercorrências. Os nove casos *single-port* foram operados por um mesmo cirurgião, no Hospital Israelita Albert Einstein. Os dados analisados das pacientes foram idade, índice de massa corporal, cirurgias anteriores e diagnóstico clínico. Os dados relacionados à cirurgia foram tempo operatório, incisão da pele, registro de complicações intraoperatórias, necessidade de conversão para laparotomia, necessidade de transferência para unidade de terapia intensiva, necessidade de transfusão sanguínea, lesão inadivertida de outros órgãos, tempo de internação e óbito.

**Resultados:**

Todos os casos foram concluídos sem intercorrências com a plataforma da Vinci Single-Site^^®^^. Quatro pacientes apresentavam adenomiose como indicação cirúrgica, duas apresentavam mioma uterino, uma câncer de endométrio, um pólipo endometrial e uma hidrorreia. A média de idade das pacientes foi 44 anos (variando de 40 a 54 anos) e o índice de massa corporal variou entre 23,4 a 33,2kg/m^2^ (média de 26,4). Nenhum caso teve qualquer tipo de complicação, como lesão intestinal ou vesical, sangramento ou necessidade de reabordagem cirúrgica. Todos os nove procedimentos foram concluídos com o portal único robótico, e nenhuma paciente necessitou de transfusão sanguínea.

**Conclusão:**

Apesar deste trabalho apresentar apenas uma série inicial de pacientes operadas por portal único robótico, ele demonstra a factibilidade do método e indica a possibilidade futura de adotar esta técnica em histerectomias eletivas e em outros procedimentos ginecológicos, assim como descrito em grandes centros de referência em cirurgia avançada no mundo. Especificamente na prática ginecológica, a evidência existente sobre o uso de portal único robô-assistido parece ser promissora e, ainda que nem todos os casos tenham indicação, é necessário que exista esta opção no arsenal cirúrgico. No entanto, estudos clínicos aleatorizados e controlados são necessários, a fim de se estabelecer a superioridade da cirurgia robótica por portal único diante da cirurgia laparoscópica com incisão única e da cirurgia laparoscópica convencional.

## INTRODUCTION

Minimally invasive surgery is the gold-standard treatment for many gynecologic diseases. Various studies have shown the laparoscopic and robotic approaches for diverse gynecological conditions are therapeutically appropriate, and improve the patients’ quality of life with surgical results equal to or better than laparotomy.^(^ ^1^ ^-^ ^6^ ^)^

Despite the potential for excellent results with laparoscopic gynecologic surgery, it is not exempt of risks, and recent reports suggest that there is a greater risk of morbidity associated with multiple incisions for the insertion of trocars, including pain, infection, and incisional hernia. In a retrospective analysis of 317 women submitted to total laparoscopic hysterectomy, 5% of pain was described at the incision sites.^(^ ^7^ ^)^

Single-incision laparoscopic surgery (SILS) is a recent technological advancement in minimally invasive surgery, developed as an even less invasive alternative than conventional laparoscopy.^(^ ^8^ ^)^ An access path to the abdominal cavity is made by means of a single incision approximately 2.5cm long, enabling performance of laparoscopic surgery with no need for multiple punctures. Many studies demonstrated that in the hands of experiences surgeons, it is viable and safe for a variety of gynecologic indications.^(^ ^9^ ^,^ ^10^ ^)^

The first laparoscopic single-port hysterectomy laparoscopic was performed by Langebrekke et al., in 2009.^(^ ^4^ ^)^ Despite the promising results published in the literature, significant difficulties were reported for this approach, such as loss of operative field, reduction in the range of movement of surgical instruments, and collisions of forceps.^(^ ^4^ ^)^

The robotic single-port system - developed over the last years, does the automatic inversion of instruments, allowing more ample movements and better ergonomy when compared to non-robotic single-port laparoscopic surgery.^(^ ^11^ ^,^ ^12^ ^)^ In addition to the three-dimension visualization, positioning of the surgeon on the robot console and the precise dissection of the anatomical structures result in a more accurate operation, with no collision of forceps. The initial studies demonstrated that this technique is also safe and effective, and can help resolve the technical limitations found in laparoscopy.^(^ ^12^ ^,^ ^13^ ^)^

A robotic surgery presents a stable optic piece, movement of the arms with the forceps connected by computerized inversion, and instruments that provide a high degree of freedom.^(^ ^12^ ^,^ ^14^ ^,^ ^15^ ^)^ Four robotic single-port hysterectomies were performed by Fader et al., in 2009, with a mean operative time of 65 minutes; the mean age and body mass index (BMI) of patients were 47 years and 28kg/m^2^, respectively. All procedures were successfully conducted by a single incision and with no postoperative complications.^(^ ^11^ ^)^

## OBJECTIVE

To present the viability of an initial series of cases of hysterectomy using the robotic single-port system at a Brazilian hospital.

## METHODS

From November 2014 to October 2016, in an unprecedented manner in Brazil, 11 patients had an initial indication and nine of them were submitted to single-port hysterectomy, using da Vinci Single-Site^®^ platform. In two patients, due to multiple prior abdominal surgeries, large uterine volume and/or uterus with no mobility, pneumoperitoneum was performed with a Verres needle and inspection of the pelvic cavity with a 5mm optics. In these cases, single-port surgery was not feasible, and robotic multiport technique was used with no intercurrent events.

The nine single-port cases were operated on by the same surgeon at *Hospital Israelita Albert Einstein.* The patients’ data analyzed included age, body mass index, prior operations, and clinical diagnosis. The data related to surgery were operative time, skin incision, record of intraoperative complications, need to convert to laparotomy, need to transfer the patient to intensive care unit (ICU), need for blood transfusions, unintended lesions of other organs, length of hospital stay, and death. The Ethics Committee approved the study with official opinion number 1.705.925, CAAE: 55828716.7.0000.0071. All participants signed an Informed Consent Form in order to voluntarily participate in the study.

### Surgical technique

**Figure 1:**
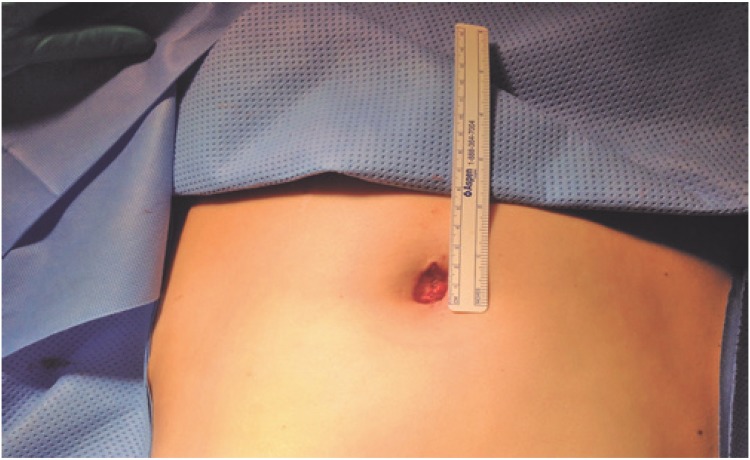
A 2.5cm umbilical incision to dissect the peritoneal cavity

Robotic single-port hysterectomy was performed through da Vinci Single-Site^^®^^, with the patient placed in a semigynecologic position under general anesthesia, with her arms duly placed along the body. A uterine manipulator and an indwelling catheter (Foley catheter) were used in all patients. An umbilical incision of 2.5cm was made in seven out of nine patients (Figure 1); in two patients, median incisions were used on prior supraumbilical scars. This was followed by dissection until the peritoneal cavity. The da Vinci Single-Site^^®^^ port was then inserted and the pneumoperitoneum was initiated, inflating up to 14mmHg. The patient's body was placed on Trendelenburg position, and the robot was placed between her legs. After introducing the camera, the trocars were inserted under direct view, and then docking (coupling of the robot) was conducted.

**Figure 2:**
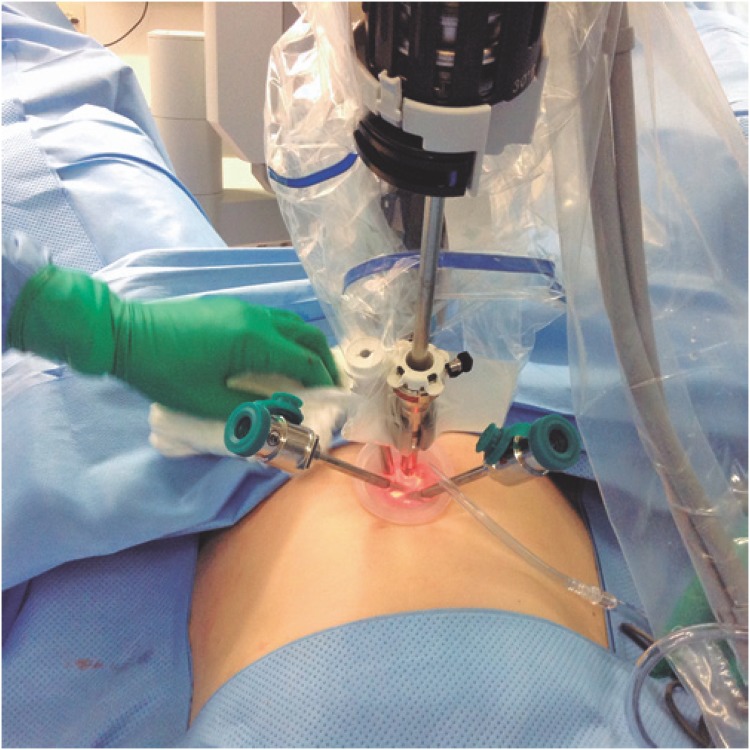
A specific four-channel port through which a three-dimension 8.5mm optics, two 5mm robotic forceps, and one 5 to 10mm forceps were introduced by the assistant

Exposure of the pelvis was reached, retracting the small bowel and sigmoid out of the pelvis. The following surgical instruments were used: a specific four-channel port through which the three-dimension 8.5mm optics was introduced, a 5mm bipolar fenestrated forceps, and a 5mm monopolar hook (Figure 2). In addition to these forceps, the assistant surgeon kept exchanging the grasping forceps with the aspirator through a 5 to 10mm passage in the same port.

In each patient, the hysterectomy technique was used according to diagnosis and anatomical conditions. On each side, the round ligament was coagulated and cut, and the retroperitoneal space was dissected to identify the ureter. Equally, on each side, the infundibulopelvic or suspensory ligament was coagulated and cut, followed by dissection of the vesicouterine pouch, and then, the uterine arteries were coagulated and. Hemostasis was performed with a bipolar forceps or monopolar hook.

In our series of cases, closing of the vaginal vault was performed by conventional technique since the articulated needle-holder that enables closing by singleport robotic method was not yet available in Brazil.

At the end of the procedure, the robotic instruments and the camera were removed, and the robot was undocked. The umbilical incision was closed by layers, and the synthesis of the skin was done with intradermal sutures.

## RESULTS

All cases were concluded with no complications using da Vinci Single-Site^®^ platform (Table 1). Four patients presented with adenomyosis as a surgical indication, two presented with uterine myoma, one with endometrial cancer, one with an endometrial polyp, and one with desquamative inflammatory vaginitis. The mean age of patients was 44 years (range of 40 to 54 years) and the BMI varied between 23.4 and 33.2kg/m^2^ (mean of 26.4). None of the cases had any type of complication, such as intestinal or bladder lesion, bleeding, or the need for a second operation. All nine procedures were concluded with the robotic single-port method, and no patient required blood transfusion. The operative results are shown on tables 2 and 3.

**Table 1:** Clinical data of nine patients submitted to robotic single-port hysterectomy

Patients	Age	BMI	Clinical complaints	Pathological result
1	42	23.4	Menorrhagia + dysmenorrhea	Adenomyosis
2	41	23.5	Hypermenorrhagia + dyspareunia	Adenomyosis + endometriosis nodule
3	51	32.3	Menorrhagia + dysmenorrhea	Myomatosis + adenomyosis
4	40	25.2	Hypermenorrhea	Adenomyosis
5	40	20.8	Menorrhagia + dysmenorrhea	Myomatosis
6	54	26.6	Postmenopausal desquamative inflammatory vaginitis	Myomatosis + isthmiccervical polyp + hydrosalpinx
7	42	33.2	Hypermenorrhea	Adenomyosis + myomatosis + endocervical polyp
8	55	25.7	Postmenopausal bleeding	Endometrioid endometrial adenocarcinoma
9	54	24.5	Postmenopausal bleeding	Myomatosis

BMI: body mass index.

**Table 2:** Operative data of nine patients submitted to robotic single-por hysterectomy

Patients	Operation performed	Weight of removed uterus (g)	Length of stay (days)
1	TH + BS	144	3
2	TH + BS + exeresis of retrocervical nodule	78	3
3	TH + BS	156	6
4	TH + BS	130	2
5	TH + BS	264	2
6	TH + BS	84	3
7	TH + BS + cholecystectomy	119	3
8	TH + SBO	123	3
9	TH + SBO	126	3

TH: total hysterectomy; BS: bilateral salpingectomy; BSO: bilateral salpingo-oophorectomy

**Table 3:** Operative data of nine patients submitted to robotic single-port hysterectomy, with the mean of the variables

Variables	Values
Total operative time, mean	132 (100-166) minutes
Length of hospital stay, mean	75 (48-144) hours
Weight of the removed uterus, mean	139 (78-264) g

## DISCUSSION

During the last decade, minimally invasive surgery, including robot-assisted surgery, has been established as a new option in the standard of surgical treatment in gynecologic diseases.^(^ ^12^ ^)^ Although some argue that there is the disadvantage of the longer operative time and higher cost, this new method is characterized by precision in surgical steps, as well as by similar or eventually better results as to blood loss, recovery time, complications, and patient comfort.^(^ ^13^ ^)^

Jung et al.,^(^ ^16^ ^)^ showed, in a literature review, that robotic single-port hysterectomy is a safe technique, with excellent esthetic results and patient satisfaction, similar to the four-port technique.

Lee et al.,^(^ ^17^ ^)^ performed robot-assisted hysterectomies with a single-port in 21 patients with uterine myomata. The mean operative time and mean blood loss were 100 minutes and 100mL, respectively.

Initially introduced in the United States about three years ago, the robotic platform da Vinci Single-Site^^®^^ was first used in Brazil, in 2014. In this project, we present the first case series of robotic single-port hysterectomies conducted in Brazil. According to the initial results, the procedure seems safe and feasible, and it is concluded without conversions and without postoperative complications. However, one should pay attention to the indication and limits of the technique, since in our collection of cases, 11 patients had indications for the procedure, but in two of them, after initiating pneumoperitoneum and inspection of the pelvic cavity, the choice for multiportal robotic surgery was made due to reduced uterine mobility and multiple adhesions.

The advantages of the robotic single-port are better esthetic results, decreased postoperative pain due to a small single incision, three dimensional visualization of the anatomical structures, stability of the instruments by the robotic platform, precision in dissections, and greater facility for the surgeon to conclude these dissections made difficult by single-port laparoscopy without a robot. Additionally, the robotic curved semi-rigid instruments favor a safe platform for the performance of the procedures and surpass the restrictions and limitations when compared to single-port conventional laparoscopy.^(^ ^12^ ^)^ It is important to point out that in patients with prior abdominal operations, especially median periumbilical, one can use this surgical scar to make a single-port.

Despite this project presenting only one initial series of patients operated by robotic single-port surgery, it demonstrates the feasibility of the method and indicates the future possibility of adopting this technique in elective hysterectomies and in other gynecologic procedures, as described in large reference centers of advanced surgery worldwide.^(^ ^8^ ^)^ Specifically in gynecological practice, the evidence of use of robot- assisted single-port surgery seems promising, and even if not all cases have an indication for it, it is important to have this option in the surgical armamentarium. Nevertheless, randomized and controlled clinical studies are required to establish the superiority of robotic single-port surgery as compared to single incision and conventional laparoscopic surgery.

## CONCLUSION

In gynecologic practice, the existing evidence on the use of a robot-assisted single-port seems promising; even if not all cases have indication for it, it is necessary to have this option in the surgical armamentarium.
